# Diagnostic Ureteroscopy for Upper Tract Urothelial Carcinoma is Independently Associated with Intravesical Recurrence after Radical Nephroureterectomy

**DOI:** 10.1590/S1677-5538.IBJU.2015.0366

**Published:** 2016

**Authors:** Pei Liu, Xiao-hong Su, Geng-Yan Xiong, Xue-Song Li, Li-Qun Zhou

**Affiliations:** 1 Department of Urology, Peking University First Hospital, Institute of Urology, Peking University, National Urological Cancer Center, Beijing, China

**Keywords:** Ureteroscopy, Oncology, Bladder, Ureter, Urinary Tract

## Abstract

**Purpose:**

To determine the effect of diagnostic ureteroscopy on intravesical recurrence in patients with upper tract urothelial carcinoma (UTUC) after radical nephroureterectomy (RNU).

**Materials and Methods:**

We conducted a retrospective analysis of 664 patients who were treated with RNU for UTUC from June 2000 to December 2011, excluding those who had concomitant/prior bladder tumors. Of the 664 patients, 81 underwent diagnostic ureteroscopy (URS). We analyzed the impact of diagnostic ureteroscopy on intravesical recurrence (IVR) using the Kaplan-Meier method. Univariate and multivariate analyses were used to determine the independent risk factors.

**Results:**

The median follow-up time was 48 months (interquartile range (IQR): 31-77 months). Patients who underwent ureteroscopy were more likely to have a small (p<0.01), early-staged (p=0.019), multifocality (p=0.035) and ureteral tumor (p<0.001). IVR occurred in 223 patients during follow-up within a median of 17 months (IQR: 7-33). Patients without preoperative ureteroscopy have a statistically significant better 2-year (79.3%±0.02 versus 71.4%±0.02, p<0.001) and 5-year intravesical recurrence-free survival rates (64.9%±0.05 versus 44.3%±0.06, p<0.001) than patients who underwent ureteroscopy. In multivariate analysis, the diagnostic ureteroscopy (p=0.006), multiple tumors (p=0.001), tumor size <3cm (p=0.008), low-grade (p=0.022) and pN_0_ stage tumor (p=0.045) were independent predictors of IVR.

**Conclusions:**

Diagnostic ureteroscopy is independently associated with intravesical recurrence after radical nephroureterectomy.

## INTRODUCTION

Upper tract urothelial carcinoma (UTUC) is rare and accounts for only 5-10% of urothelial carcinoma cases, with an annual incidence of 1-2 cases per 100.000 in Western countries (1–3). Radical nephroureterectomy (RNU) with bladder cuff excision is the gold standard for managing UTUC (4). The incidence of subsequent intravesical recurrence (IVR) following RNU is 22-47%, which means a close follow-up using cystoscopy is required to detect the high incidence of IVR (5, 6).

Several risk factors are reported to be associated with IVR, such as the age, sex, tumor location, multiplicity, size, surgical approach (open or laparoscopic surgery) and distal ureter management, T stage, grade, carcinoma in situ, and history of bladder cancer (7). With the development of medical devices, diagnostic ureteroscopy is becoming a powerful tool for patients with UTUC, which could contribute to the diagnostic certainty and decision making regarding treatment options. The European guidelines suggest that diagnostic ureteroscopy should be performed in the preoperative assessment of any UTUC patient (Grade C) (8). Concerns, however, have been raised that there is a possible risk of tumor implantation during ureteroscope manipulation and irrigation (9).

To the best of our knowledge, previous studies are sparse, and there is no consistent conclusion on the impact of diagnostic ureteroscopy for intravesical recurrence (9–11). Therefore, we conducted this study, based on data from a large center in China, to determine whether diagnostic ureteroscopy results in IVR after RNU.

## MATERIALS AND METHODS

The follow-up data of patients with UTUC who were treated with RNU at Peking University First Hospital, Beijing, China, from June 2000 to December 2011 were reviewed. Out of 892 patients submitted to RNU in our service we selected 753 with complete FU information. Among these 753 patients, we excluded 82 patients with concomitant/prior bladder tumors, 6 patients with bilateral UTUC, and 1 patient with distant metastasis. Therefore, 664 patients were included in this study. None of these patients received neoadjuvant or adjuvant chemotherapy.

Of 664 patients, 583 in the control group were diagnosed by CT/MRI, urinary cytology specimens and retrograde pyelography. Eighty-one patients underwent diagnostic ureteroscopy and were included in the study group. Diagnostic ureteroscopy is especially used when there is diagnostic uncertainty or when conservative treatment is being considered, but there are no standardized prospective criteria. Chest X-ray and preoperative cystoscopy were performed in all patients to rule out metastasis and concomitant bladder tumors.

All patients underwent retroperitoneal open or laparoscopic nephroureterectomy. In all cases, the ureter was ligated immediately after control of the renal artery without dissecting around the kidney. The distal ureter and bladder cuff were all managed by extravesical dissection and the intramural portion within the bladder wall through an open Gibson incision. A regional lymph node dissection was performed in case of suspicious lymph node invasion on preoperative imaging or intraoperative examination.

The clinicopathologic data were retrospectively recorded. All tumors were graded by the World Health Organization classification of 2004 and staged by the Union for International Cancer Control TNM classification of malignant tumors 2002. The tumor location was defined as renal pelvis or ureter, and tumor multifocality was defined as the presence of two or more macroscopic tumors in the upper urinary tract.

During follow-up, patients received cystoscopy every 3 months for the first 2 years, which extended to 1 year thereafter. Serum creatinine level, chest X-ray and CT or MRI were performed simultaneously.

All statistical data were managed with SPSS version 19.0. Statistical significance was set at p<0.05. Continuous variables were compared using the two-sample t-test, and categorical variables were compared using the Chi square test. The Kaplan-Meier method was used to estimate the survival outcomes. Univariate analysis with the log-rank test and multivariate analysis with Cox proportional hazards regression model were used. Only variables that were significant according to univariate analysis were considered for the multivariate analysis.

## RESULTS

The clinicopathologic features are summarized in Table-1. The median patient age was 68 years (IQR: 60-74 months). The median follow-up time was 48 months (IQR: 31-77 months). There was no significant difference in gender, age, smoking status, surgical mode, presence of hydronephrosis, presence of carcinoma in situ, N stage or tumor grade. Patients who underwent ureteroscopy were more likely to have a small (p<0.01), early-staged (p=0.019), multifocality (p=0.035) and ureteral tumor (p<0.001).

**Table 1:** Clinicopathological characteristics of 664 patients with RNU for UTUC.

		URS(+) n=81	URS(-) n=583	p values
**Gender**				
	Male	31(38.3%)	264(45.3%)	0.234
	Female	50(61.7%)	319(54.7%)	
**Age (years)**		65.9±11.3	66.6±10.7	0.617
**Smoking**				
	yes	10(12.3%)	111(19.0%)	0.144
	no	71(87.7%)	472(81.0%)	
**Surgical mode**				
	Laparoscopic	23(28.4%)	196(33.6%)	0.349
	Open	58(71.6%)	387(66.4%)	
**Tumor location**				
	Pelvis	26(32.1%)	342(58.7%)	P<0.001*
	Ureter	55(67.9%)	241(41.3%)	
**Multifocality**				
	Multiple	27(33.3%)	132(22.6%)	0.035*
	Solitary	54(66.7%)	451(77.4%)	
**Hydronephrosis**				
	present	45(55.6%)	329(56.4%)	0.882
	absent	36(44.4%)	254(43.6%)		
**Tumor size**				
	≥3 cm	28(34.6%)	335(57.5%)	P<0.001*
	<3 cm	53(65.4%)	248(42.5%)	
**Cis**				
	present	4(4.9%)	15(2.6%)	0.400
	absent	77(95.1%)	568(97.4%)	
**pT stage**				
	<T2	65(80.2%)	393(67.4%)	0.019*
	>T2	16(19.8%)	190(32.6%)	
**N stage**				
	N+	3(3.7%)	44(7.5%)	0.206
	N0	78(96.3%)	539(92.5%)	
**Tumor grade**				
	Low	51(63.0%)	330(56.6%)	0.278
	High	30(37.0%)	253(43.4%)	

* p<0.05, significant difference was reached

**Figure 1:**
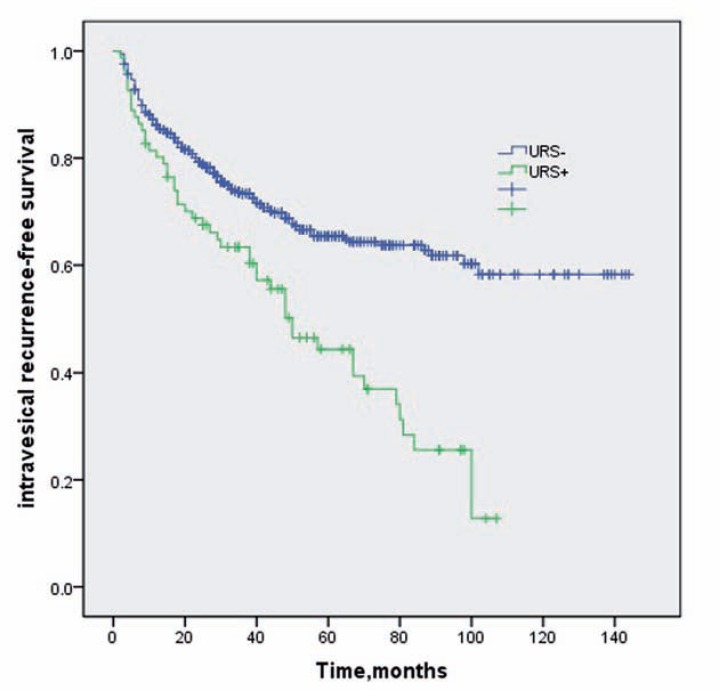
Kaplan-Meier survival curves for intravesical recurrence-free survival stratified by preoperative diagnostic ureteroscopy.

There was intravesical recurrence in 223 patients (33.6%) during follow-up within a median of 17 months (IQR: 7-33 months). The 2-year and 5-year intravesical recurrence-free survival rates for patients with and without ureteroscopy were 71.4%±0.02 versus 79.3%±0.02 and 44.3%±0.06 versus 64.9%±0.05, respectively (Figure-1).

In univariate analysis, diagnostic ureteros-copy and tumor multifocality, tumor size, tumor location, T stage, N stage and grade were all associated with intravesical recurrence while in multivariate Cox regression analysis only the diagnostic ureteroscopy (p=0.006), multiple tumors (p=0.001), tumor size <3cm (p=0.008), low-grade tumor (p=0.022) and pN_0_ stage (p=0.045) were independent predictors (Table-2).

**Table 2:** Univariable and multivariate Cox regression analyses predicting intravesical recurrence in 664 patients after nephroureterectomy for UTUC.

	Univariate analysis (P value)	Multivariate Cox Regression analysis		
		HR	95% CI	P
**Diagnostic Ureteroscopy**	<0.001	1.592	1.143-2.218	0.006*
**Multifocal tumor**	<0.001	1.596	1.206-2.111	0.001*
**Tumor size < 3 cm**	<0.001	1.459	1.104-1.929	0.008*
**Low-grade tumor**	0.001	1.438	1.053-1.964	0.022*
**pN_0_ stage**	0.004	2.512	1.019-6.195	0.045*
**Tumor located in ureter**	0.01	1.295	0.984-1.705	0.065
**pT stage** ≤ **T_2_**	0.023	0.907	0.639-1.287	0.982
**Age more than 70**	0.737			
**Presence of hydronephrosis**	0.065			
**Smoking**	0.934			
**Laparoscopic surgery**	0.871			
**Female**	0.293			
**Presence of Cis**	0.606			

* p<0.05, significant difference was reached

## DISCUSSION

RNU with bladder cuff excision is the gold standard for managing UTUC (4). However, a high potential of intravesical recurrence after RNU has been reported. In the guidelines for NCCN, all UTUC patients after RNU are recommended to undergo cystoscopy for routine bladder surveillance (12). In this retrospective study, the incidence of IVR was 33.6%, which is in agreement with previous reports (22-47%) (5, 6). The explanation of such a high incidence of IVR can only be hypothesized, including the field cancerization hypothesis and intraluminal seeding of the tumor (13, 14).

Several studies have evaluated the risk factors for IVR after RNU, such as age, gender, tumor multiplicity, TNM stage, grade, tumor location, size, previous/concomitant bladder tumors, carcinoma in situ, surgical mode and distal ureter management (7, 15–22). Among these factors, a history of a prior bladder tumor and a multifocal primary tumor are the most frequently reported, while others are still under debate. In the present study, however, we excluded the patients with previous/concomitant bladder cancer because the incidence of IVR in those patients is related to local disease instead of UTUC or ureteroscopic procedure. The presence of multiple tumors remains the predictive factor for IVR when patients with a history of bladder cancer are excluded. Additionally, low-grade tumors, pN_0_ stage and tumor size <3cm are associated with IVR. In our previous study (7), the influence of tumor grade on bladder recurrence was not that significant when excluding the patients that died during follow-up without bladder recurrence (p=0.061). Thus, we consider this result may be attributed to the fact that high-grade, N_+_ patients or those with large tumors may suffer from tumor dissemination and die prior to the detection of IVR.

With the development of medical devices, diagnostic ureteroscopy is becoming a powerful tool for patients with UTUC, and it is used to visualize and biopsy the entire upper urinary tract with a technical success approaching 95% (23). Coupled with biopsies, it provides satisfactory diagnostic accuracy (24). However, concerns have been raised that tumor implantation may result from ureteroscope manipulation and irrigation. Retrograde flow, increased urine flow rate and intraluminal pressure might lead to the shedding of tumor cells, which implant in the bladder to develop recurrences. There are only 3 previous studies on the impact of diagnostic ureteroscopy for IVR after RNU, and they do not reach a consistent conclusion (9–11). Nison et al. reported that there was intravesical recurrence 146 times, which was 28% in the URS group and 27.5% in the URS+group (not significantly different). In addition, they did not find that ureteroscopy is an independent risk factor for IVR (9). Ishikawa et al. reached the same conclusion as Nison, and the 2-year bladder recurrence-free survival rate was 60.0% in their URS+group and 58.7% in their control group (10). Luo et al., by contrast, reported that ureteroscopy was associated with an increased incidence of intravesical recurrence in patients with or without a history of bladder cancer (11). In the present study, it is possible that diagnostic ureteroscopy is an independent predictive factor for IVR, providing evidence for the intraluminal seeding hypothesis.

The question remains whether we should use diagnostic ureteroscopy as a routine preoperative examination. The European guidelines suggest that diagnostic ureteroscopy should be performed in the preoperative assessment of any UTUC patient (8). However, diagnostic ureteros-copy may have the potential risk of future intravesical recurrence by increasing tumor cell shedding during manipulation and irrigation. We should find a balance between misdiagnosis without preoperative URS, which may result in unnecessary nephrectomy, and the potential risk of IVR after RNU.

There are several limitations of this study, including the retrospective design and data collection, which may lead to selection and recall bias. No strict criteria for preoperative ureteroscopy were established, and each surgeon independently made the decision to perform URS. In spite of these limitations, our study is still the largest single-center study in China on IVR after RNU for patients without a history of bladder cancer. Further molecular genetic studies and randomized control trials are needed to help determine the mechanism for intravesical recurrence.

## CONCLUSIONS

Diagnostic ureteroscopy is independently associated with intravesical recurrence after radical nephroureterectomy. Urologists should reconsider the use of diagnostic ureteroscopy as a routine preoperative assessment. We should find a balance between misdiagnosis without preoperative URS, which may result in unnecessary nephrectomy, and the potential risk of IVR after RNU.

## ABBREVIATIONS

UTUC: = Upper tract urothelial carcinoma
RNU: = Radical nephroureterectomy
IVR: = intravesical recurrence
